# Association between omentin and obstructive sleep apnea: A meta‐analysis

**DOI:** 10.1111/crj.13589

**Published:** 2023-01-31

**Authors:** Ningning She, Na Liu, Xiaoyong Ren, Haiqin Liu

**Affiliations:** ^1^ Department of Otorhinolaryngology The Second Affiliated Hospital of Xi'an Jiaotong University Xi'an China

**Keywords:** adipokine, meta‐analysis, obstructive sleep apnea, omentin

## Abstract

**Background:**

Obstructive sleep apnea (OSA) can lead to multisystem and multiorgan damage, which has attracted widespread attention from scholars. The pathogenesis of OSA is complex, and obesity plays an important role. Adipokine is secreted by adipose tissue, and its abnormal expression may be closely related to OSA. The relationship between omentin (a novel adipokine) and OSA is controversial. This study focuses on the important role of omentin in OSA and explores whether it can be regarded as a new target for the diagnosis and treatment of OSA.

**Method:**

PubMed, Embase, Web of Science, the Cochrane library, WANFANG, VIP, and Chinese National Knowledge Infrastructure were systematically searched for retrieving eligible studies until May 2022. Documents were screened according to strict inclusion and exclusion criteria, and data were extracted using Excel spreadsheets. The quality of the literature was assessed using the Newcastle–Ottawa Scale. RevMan 5.3 and Stata 12.0 software were used in this meta‐analysis for data synthesis.

**Result:**

A total of eight eligible studies with 23 databases involving 914 participants were included in this meta‐analysis. Combined data indicated that omentin levels in OSA patients were lower than that in controls (standardized mean difference = −1.54, 95% confidence interval = −2.07 to −1.00, *p* < 0.001). According to the subgroup analysis results of different races, sample source, gender, and the severity of the disease, compared with that in the control group, the level of omentin in OSA patients was significantly lower. When conducting sensitivity analysis, the results of the study were less stable. Meta‐analysis indicated that there was no publication bias in this study. The omentin levels were significantly lower in OSA patients. The findings suggest that omentin may be a potential marker for the diagnosis and treatment of OSA. However, the heterogeneity of this study is high, and more high‐quality large‐sample studies will be needed in the future.

## Graphical abstract

This study used a meta‐analysis method to explore the expression of serum omentin in patients with obstructive sleep apnea (OSA). The results found that the level of omentin in OSA patients was significantly reduced, suggesting that omentin may play a role in the pathogenesis of OSA, and it is worthy of further study as a potential marker of OSA.

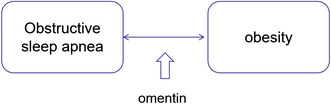

## 1 INTRODUCTION

Obstructive sleep apnea (OSA) is caused by partial or complete upper airway obstruction during nighttime sleep, characterized by repeated hypoxemia and/or apnea at night. The clinical manifestations are snoring at night, drowsiness during the day, inattentiveness, and a series of multisystem dysfunction, which can easily lead to complications such as hypertension, diabetes, cardiovascular, and cerebrovascular diseases.^1^, ^2^, ^3^ The pathogenesis of OSA is complex and diverse, and scholars have found that obesity may be an important confounding factor in OSA. The incidence of OSA in obese patients can reach more than 40%, and the incidence of obesity in OSA patients can reach 75.2%.^4^ Obesity causes abnormal distribution and excessive accumulation of fat. Furthermore, abdominal visceral adipose accumulation was an independent risk factor for OSA.^5^ Recent studies have found that adipose tissue is a very active endocrine organ that secretes a variety of biologically active signaling molecules collectively referred to as adipocytokines or adipokines.^6^, ^7^

Omentin is a newly discovered “beneficial” adipokine, which has different subtypes. Omentin‐1 is a protein hormone specifically expressed in omental adipose tissue. It has important functions such as anti‐inflammatory, anti‐atherosclerosis, regulating fat metabolism, and improving insulin sensitivity.^8^, ^9^ Scholars have found that it is closely related to insulin resistance, diabetes, metabolic syndrome, and inflammatory response.^10^ Omentin‐1 is also considered to be an important hub connecting OSA and its complications such as diabetes, hypertension, obesity, and atherosclerosis.^11^ However, the association between omentin‐1 and OSA is controversial. Previous related clinical researches were mostly single clinical trials. Due to the limitations of the research objects and research conditions, there were obvious limitations between the researches, and the research results were not uniform.

Therefore, in this study, we used a meta‐analysis to evaluate the association between omentin and OSA. From the perspective of evidence‐based medicine, the aim of this study is to provide new ideas for the clinical diagnosis and treatment of OSA and the related mechanisms of complications.

## 2 METHODS

This meta‐analysis is being reported in accordance with Preferred Reporting items for Systematic Reviews and Meta‐analysis statement.^12^

### 2.1 Literature sources and search strategy

PubMed, Embase, Web of Science, the Cochrane library, WANFANG (Chinese database), VIP (Chinese Database), and Chinese National Knowledge Infrastructure were systematically searched for retrieving eligible studies until May 2022. Keywords and search strategy were as follows: “obstructive sleep apnea hypopnea syndrome” or “OSAHS” or “obstructive sleep apnea” or “OSA” or “obstructive sleep apnea syndrome” or “OSAS” or “obstructive sleep hypopnea” or “sleep apnea” combined with “omentin.” In addition, to minimize the omission of literature, an independent supplementary manual search was performed on the reference lists of retrieved articles.

### 2.2 Study selection

Inclusion criteria are the following: (1) The study design was a case–control correlation analysis which must have reported values in mean and standard deviation or median with range of adiponectin levels in individuals with OSA and without OSA; (2) OSA was defined as apnea hypopnea index (AHI) ≥ 5; and (3) all OSA patients were diagnosed for the first time, without receiving any form of treatment.

Exclusion criteria are the following: (1) duplicate publication of articles; (2) review and editorial articles, case reports, animal experiments, and conference abstracts; and (3) original papers that did not contain precise data about levels of omentin in patients or controls.

### 2.3 Data extraction

The study data were independently and blindly extracted by two researchers (She Ningning and Na Liu), and in case of disagreement, a third party (Xiaoyong Ren) was invited to judge. A standardized excel form was used to extract relevant data from all studies, including first author's name, publication year, population country, total sample size, the source of omentin, omentin levels in patients or controls, age, gender, and body mass index (BMI).

### 2.4 Quality assessment

The quality of the included literature was assessed using the Newcastle–Ottawa Scale grading system by two researchers (Ningning She and Na Liu). In case of disagreement, it will be assessed by a third‐party reviewer (Xiaoyong Ren).

### 2.5 Statistical analysis

Statistical analyses were performed by using Review manager 5.3 and STATA version 12.0. Due to the inconsistency of measurement units and assay approaches, standardized mean difference (SMD) with 95% confidence intervals (CIs) was chosen as effect size. Statistical heterogeneity was assessed using *I* ^2^ metric statistics. The fix‐effects model was used in case of no substantial statistical heterogeneity; otherwise, the heterogeneity was evaluated using the random‐effects model. An *I* ^2^ of 25% to 49% was considered to represent a low level of heterogeneity, 50% to 74% a moderate level, and 75% to 100% a high level. To explore the sources of heterogeneity, we performed subgroup analysis by race, age, sample source, BMI, gender, and severity of illness. Sensitivity analysis was conducted to evaluate the stability of pooled results. Begg's funnel plot and Egger's test were conducted to examine the underlying publication bias. *P* < 0.05 were considered statistically significant.

## 3 RESULTS

### 3.1 Study inclusion and characteristics

**FIGURE 1:**
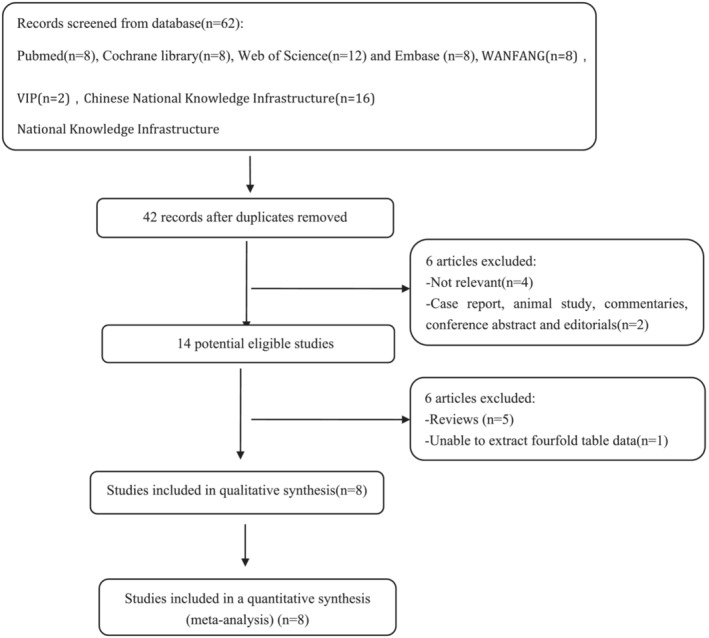
Flow diagram of screened and included papers.

Figure 1 shows the detailed process of literature inclusion and exclusion. A total of 62 papers were initially retrieved from the database, 20 duplicate papers were further eliminated, and the remaining 42 papers were screened by title and abstract. After excluding irrelevant and case studies, the remaining 14 articles were reviewed, among which five articles were reviews and one article could not obtain valid data. Finally, a total of eight studies with 23 datasets met inclusion criteria and were pooled for this meta‐analysis.^13^, ^14^, ^15^, ^16^, ^17^, ^18^, ^19^, ^20^ In our meta‐analysis, there were total involving 914 participants (OSA subjects [*N* = 588] and controls [*N* = 326]). The principal characteristics extracted from these individual studies are listed in Tables 1 and 2.

**TABLE 1:** Characteristics of included studies.

Author	Year	Country	*N* (NOR/OSA)	M/F	Sample source	NOS
Tang^17^	2019	China	95 (30/65)	95/0	Serum	7
Zhong^18^	2015	China	130 (26/104)	130/0	Serum	6
Liu^19^	2014	China	75 (30/45)	43/32	Plasma	6
Zhang^13^	2018	China	50 (20/30)	50/0	Plasma	8
Wang^16^	2013	China	336 (144/192)	336/0	Serum	7
Uygur^14^	2016	Turkey	127 (31/96)	89/38	Serum	6
Zirlik^20^	2013	Germany	20 (10/10)	19/1	Plasma	8
Kurt^15^	2014	Turkey	81 (35/46)	54/27	Plasma	7

Abbreviations: F, female; M, male; *N*, sample size; NOS, Newcastle–Ottawa Scale; NOR, control group; OSA, obstructive sleep apnea group.

**TABLE 2:** Participants' characteristics of included studies.

Author	Age (mean ± *SD*), years		BMI (mean ± *SD*), kg/m^2^		AHI (mean ± *SD*)		Omentin (mean ± *SD*), ng/ml	
	NOR	OSA	NOR	OSA	NOR	OSA	NOR	OSA
Tang (mild–mod)	44.43 ± 9.58	45.47 ± 8.78	25.88 ± 2.37	25.96 ± 3.44	3.56 ± 0.80	16.4 ± 13.05	28.28 ± 4.76	23.33 ± 5.82
Tang (sev)	44.43 ± 9.58	45.33 ± 8.45	25.88 ± 2.37	27.24 ± 2.08	3.56 ± 0.80	40.0 ± 9.45	28.28 ± 4.76	18.34 ± 6.57
Liu	47.01 ± 8.55	47.83 ± 11.08	27.98 ± 6.41	28.5 ± 6.12	1.51 ± 0.27	31.74 ± 6.13	23.91 ± 5.07	18.57 ± 4.02
Zhang	36.10 ± 13.67	40.73 ± 8.9	27.55 ± 2.87	28.85 ± 2.62	1.93 ± 1.38	61.48 ± 15	57.35 ± 22.87	44.29 ± 19.19
Wang (mild)	48.74 ± 10.62	—	27.14 ± 3.28	—	2 ± 1	—	22.62 ± 5.89	12.33 ± 2.23
Wang (mod)	48.74 ± 10.62	—	27.14 ± 3.28	—	2 ± 1	—	22.62 ± 5.89	11.98 ± 3.56
Wang (sev)	48.74 ± 10.62	—	27.14 ± 3.28	—	2 ± 1	—	22.62 ± 5.89	10.2 ± 4.11
Uygur (mild)	50.6 ± 12.8	—	29.6 ± 4.1	—	1.8 ± 1.7	—	42.5 ± 5.2	34.2 ± 8.6
Uygur (mod)	50.6 ± 12.8	—	29.6 ± 4.1	—	1.8 ± 1.7	—	42.5 ± 5.2	26.9 ± 5.4
Uygur (sev)	50.6 ± 12.8	—	29.6 ± 4.1	—	1.8 ± 1.7	—	42.5 ± 5.2	22.3 ± 7.6
Kurt (mild)	42.8 ± 14.1	50.3 ± 7.8	26.4 ± 6.8	28.1 ± 3.5	—	—	432 ± 276.8	480 ± 168
Kurt (mod)	42.8 ± 14.1	45.7 ± 15.3	26.4 ± 6.8	31.2 ± 4.7	—	—	432 ± 276.8	654 ± 359
Kurt (sev)	42.8 ± 14.1	49.3 ± 11.6	26.4 ± 6.8	34.1 ± 6.5	—	—	432 ± 276.8	549 ± 261
Zhong (normal, mild)	41.33 ± 14.23	46.44 ± 16.98	22.49 ± 1.2	22.51 ± 1.28	1.11 ± 0.93	7.56 ± 2.92	339.01 ± 34.62	338.55 ± 30.07
Zhong (normal, mod)	41.33 ± 14.23	55 ± 13.48	22.49 ± 1.2	22.17 ± 4.38	1.11 ± 0.93	20.44 ± 4.56	339.01 ± 34.62	262.68 ± 30.05
Zhong (normal, sev)	41.33 ± 14.23	54.7 ± 21.1	22.49 ± 1.2	22.13 ± 1.25	1.11 ± 0.93	45.4 ± 13.75	339.01 ± 34.62	133.01 ± 8.54
Zhong (overweight, mild)	53.22 ± 15.18	50.66 ± 18.14	25.77 ± 1.48	26.06 ± 0.74	2.56 ± 1.42	8.78 ± 2.54	275.71 ± 36.38	276.28 ± 21.25
Zhong (overweight, mod)	53.22 ± 15.18	54.72 ± 12.88	25.77 ± 1.48	26.06 ± 0.74	2.56 ± 1.42	24.63 ± 3.85	275.71 ± 36.38	234.02 ± 28.59
Zhong (overweight, sev)	53.22 ± 15.18	50.68 ± 15.61	25.77 ± 1.48	26.10 ± 1.28	2.56 ± 1.42	48.84 ± 11.98	275.71 ± 36.38	78.44 ± 15.28
Zhong (obesity, mild)	43.25 ± 13.83	49.5 ± 16.26	30.10 ± 1.93	29.55 ± 1.31	2.25 ± 0.89	9.2 ± 3.08	241.25 ± 37.5	240.22 ± 39.17
Zhong (obesity, mod)	43.25 ± 13.83	48.66 ± 14.04	30.10 ± 1.93	29.97 ± 1.24	2.25 ± 0.89	22.44 ± 5	241.25 ± 37.5	150.02 ± 33.03
Zhong (obesity, sev)	43.25 ± 13.83	49.66 ± 7.83	30.10 ± 1.93	31.34 ± 2.24	2.25 ± 0.89	60.0 ± 15.96	241.25 ± 37.5	59.63 ± 27.77
Zirlik	53.6 ± 7.7	58.9 ± 10.2	26.7 ± 2.3	31.7 ± 3.2	2.7 ± 3.1	40.4 ± 18.9	9.24 ± 4.85	17.22 ± 13.94

Abbreviations: BMI, body mass index; NOR, control group; OSA, obstructive sleep apnea; *SD*, standard deviation.

### 3.2 Assessment of study quality

We used the Newcastle–Ottawa Scale to evaluate the quality of the included studies. The quality scale consists of three parts: selection, comparability, and exposure assessment, and the quality score ranges from 0 to 9. In our meta‐analysis, all studies have scores higher than 6 stars as high‐quality studies (Table 1).

### 3.3 Pooled analysis

**FIGURE 2:**
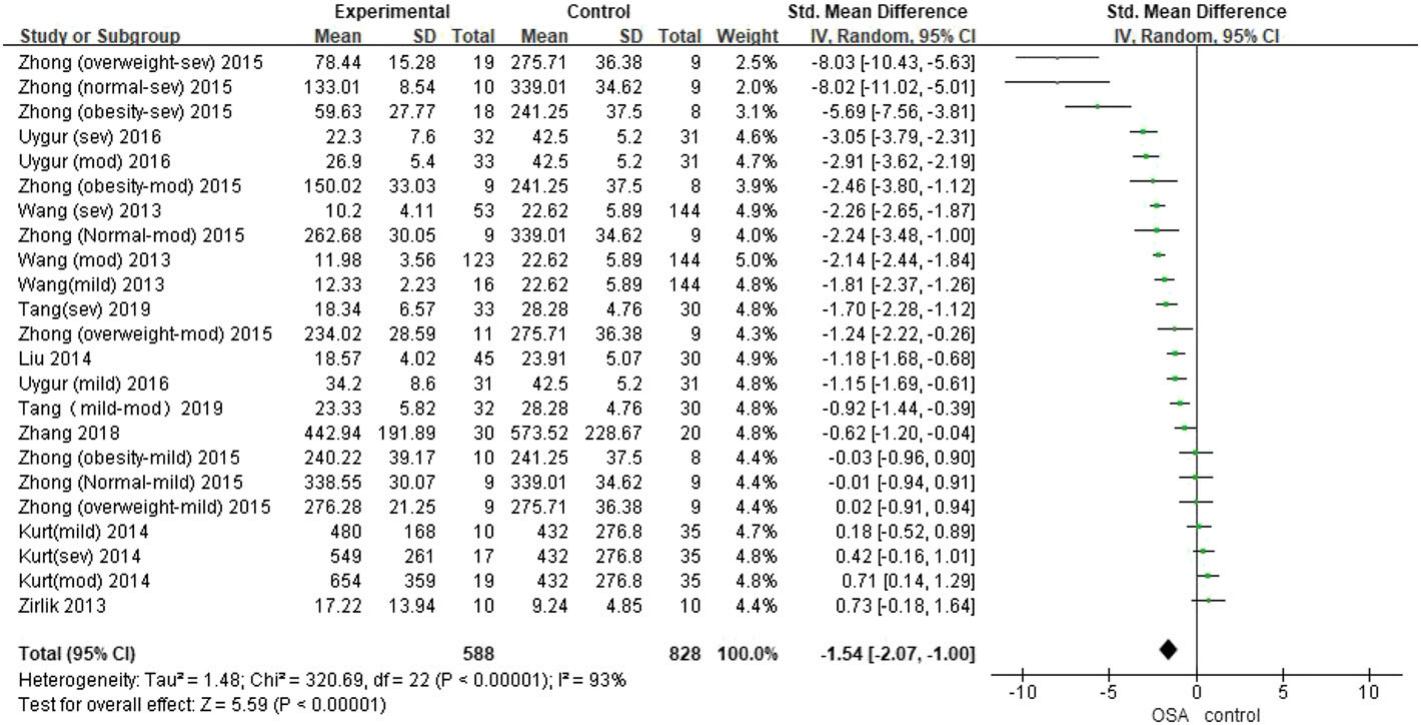
Forest plots of studies on omentin levels for obstructive sleep apnea hypopnea syndrome patients versus controls.

The value of *I* ^2^ was 93%, which indicates a high degree of heterogeneity in the findings. Therefore, we chose a random‐effects model for combining effect sizes. Meta‐analysis exhibited that omentin levels in OSA patients were significantly lower than in controls (SMD = −1.54, 95% CI = −2.07 to −1.00, *p* < 0.001) (Figure 2).

### 3.4 Subgroup analysis

In order to reduce the inhibition of the study and reduce the influence of confounding factors, the study performed subgroup analysis on the main confounding factors (race, age, sample source, BMI, gender, and severity of illness), and the results are presented in Table 3. Subgroup analysis results of different races show that in Chinese population, the difference of omentin level between OSA patients and control group is statistically significant, and the expression of OSA patients is lower (SMD = −1.98, 95% CI = −2.50 to −1.46, *p* < 0.001). When performing subgroup analysis based on sample source, in serum, the difference between the level of omentin in OSA patients and the level of control group was statistically significant, and the expression in OSA patients was lower (SMD = −2.07, 95% CI = −2.61 to −1.54, *p* < 0.001). When analyzed by gender as a subgroup, the difference between the level of omentin in OSA patients and the level of control group in a single male patient was statistically significant, and the expression of OSA patients was lower (SMD = −1.91, 95% CI = −2.50 to −1.32, *p* < 0.001). When performing subgroup analysis based on the severity of the disease, in severe OSA, the difference between the omentin level of OSA patients and the control group was statistically significant, and the expression of OSA patients was lower (SMD = −3.51, 95% CI = −4.92 to −2.10, *p* < 0.001).

**TABLE 3:** Subgroup analysis of omentin levels in obstructive sleep apnea patients and controls.

Subgroup	*N*	SMD (95%)	*Z*	*p*	Test of heterogeneity	
					*I* ^2^ (%)	*p*
Overall	23	−1.54 (−2.07, −1.00)	5.59	<0.001	93	<0.001
Race						
Chinese	16	−1.98 (−2.50, −1.46)	7.47	<0.001	90	<0.001
Not Chinese	7	−0.72 (−1.89, 0.44)	1.22	<0.001	95	<0.001
Age						
≥50	7	−2.17 (−3.76, −0.58)	2.68	0.007	93	<0.001
<50	10	−0.93 (−1.63, −0.23)	2.61	0.009	90	<0.001
Sample source						
Serum	17	−2.07 (−2.61, −1.54)	7.58	<0.001	89	<0.001
Plasma	6	0.73 (−0.66, 0.69)	0.04	0.97	86	<0.001
BMI						
≥30	4	−0.63 (−2.14, 0.89)	0.81	0.42	93	<0.001
<30	13	−1.42 (−2.07, −0.77)	4.26	<0.001	87	<0.001
Gender						
Male	15	−1.91 (−2.50, −1.32)	6.31	<0.001	90	<0.001
Male and female	8	−0.78 (−1.76, 0.20)	1.56	0.12	95	<0.001
Condition						
Mild	6	−0.52 (−1.25, 0.22)	1.38	0.17	84	<0.001
Moderate	6	−1.68 (−2.89, −0.48)	2.75	0.006	94	<0.001
Severe	7	−3.51 (−4.92, −2.10)	4.87	<0.001	95	<0.001

Abbreviations: BMI, body mass index; SMD, standardized mean difference.

### 3.5 Sensitivity analysis

**FIGURE 3:**
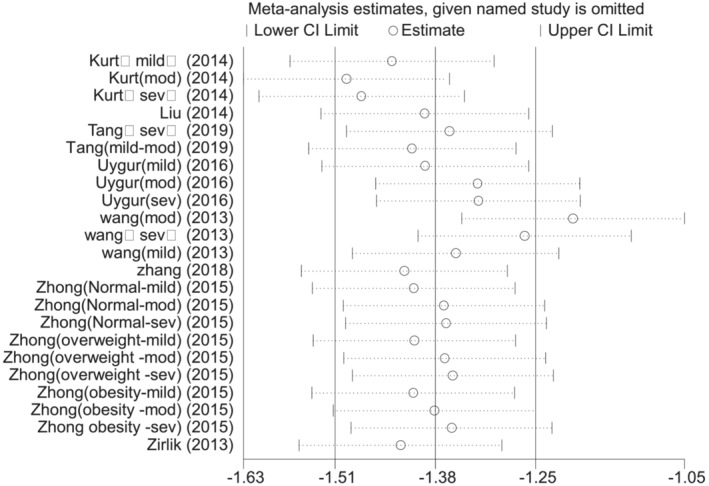
Sensitivity analysis of studies on omentin levels for obstructive sleep apnea hypopnea syndrome patients versus controls.

To explore the stability of the findings, we performed a sensitivity analysis (Figure 3). The analysis showed that deleting the study^16^ significantly changed the combined results, indicating that the results were unstable (data not shown).

### 3.6 Publication bias

**FIGURE 4:**
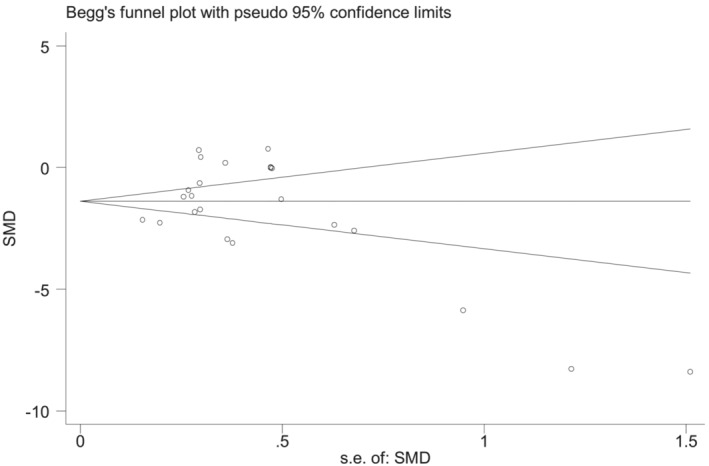
Funnel plot for all studies included in the meta‐analysis.

Although the funnel plot in the study results was not completely contralateral (Figure 4), the results of Begg's test and Egger's test were not statistically significant (*p* = 0.369, *p* = 0.757), and it can be considered that the study had no obvious publication bias.

## 4 DISCUSSION

OSA is mainly characterized by intermittent hypoxia and sleep fragmentation. Long‐term chronic inflammatory state of the body seriously affects human health and may lead to systemic organ dysfunction. The pathogenesis of OSA is affected by many factors. Obesity is considered to induce the body to be in a state of chronic inflammation and is an important risk factor for OSA. The incidence of OSA in people with obesity is as high as 30%.^21^ At present, scholars have found that changes in adipokines may be a potential mechanism for explaining the pathophysiological link between OSA and endocrine disorders such as obesity.^22^

Adipose tissue has an active endocrine function and can secrete a variety of adipokine factors. Omentin is a recently discovered new type of adipokine. Many studies have found that omentin is widely involved in regulating and maintaining the balance of sugar and lipid metabolism and vascular endothelial function in the metabolic fields such as obesity, diabetes, and cardiovascular disease. Scholars have found that omentin‐1 is associated with inflammation,^23^ which increases the expression of endothelial nitric oxide and reduces inflammation and oxidative stress.^24^ The reduction of serum omentin levels is associated with obesity, Type 2 diabetes mellitus, coronary atherosclerotic heart disease, and other diseases.^25^

Inflammatory response has been considered as a potential mechanism of OSA. Elevated levels of various circulating inflammatory markers, such as tumor necrosis factor alpha, interleukin‐6, and C‐reactive protein, have been suggested to be associated with OSA.^26^, ^27^ However, it has been found in many studies that omentin can inhibit inflammation, and serum omentin level is negatively correlated with tumor necrosis factor alpha and interleukin‐6.^8^, ^28^ So, some scholars believe that changes in the level of omentin will destroy the inflammatory response, which is also considered to be the main common way to cause and exacerbate OSA. Liu et al. found that plasma omentin‐1 level in OSA patients was negatively correlated with AHI through correlation analysis. Further multiple regression analysis showed that AHI was an independent factor affecting omentin‐1 level.^19^ Wang et al. also found that serum omentin‐1 levels were an independent determinant of the presence of OSA.^16^ Severe OSA patients had significantly decreased levels of serum omentin‐1 compared with mild and moderate OSA patients. Therefore, the levels of serum omentin‐1 could serve as a new biomarker to predict the presence and severity of OSA.

At present, most of the relevant studies carried out by scholars are single‐sample studies, which are limited by the sample data, and the results of the various studies vary greatly. Therefore, this study summarizes the published studies on the content of omentin in OSA and uses meta‐analysis to further prove the hearing impairment of OSA patients through large sample data, in order to provide new ideas for the diagnosis and treatment of OSA for clinicians.

This study is the first meta‐analysis to evaluate the relationship between omentin levels in OSA patients and control subjects. Most of the previous studies were single‐sample studies, and the number of subjects was limited, and the results were contradictory. In this study, the meta‐analysis method was used to search the database for publicly published clinical studies on omentin and OSA. After statistical analysis, a total of eight studies with 23 datasets met inclusion criteria and were pooled for this meta‐analysis; there were total involving 914 participants (OSA subjects [*N* = 588] and controls [*N* = 326]). In our meta‐analysis, all studies have scores higher than 6 stars as high‐quality studies. Meta‐analysis exhibited that omentin levels in OSA patients were significantly lower than that in controls (SMD = −1.54, 95% CI = −2.07 to −1.00, *p* < 0.001). The results of the study suggest that the expression of omentin in serum/plasma in OSA patients is significantly lower than that in the normal control group, but its specific mechanism of action is not clear. Considering the high heterogeneity of studies, in order to reduce research inhibition and reduce the interference of confounding factors, we performed subgroup analysis, and the results showed that race, sample source, gender, and disease severity may be important influencing factors leading to heterogeneity. According to the sensitivity analysis, the stability of the research results is not good, and more high‐quality literature is needed to support it. In order to improve the reliability of the results of this study, during the meta‐analysis process, strict inclusion and exclusion criteria were formulated, and two statistical researchers independently extracted data and evaluated the quality of literature in order to reduce the possibility of data extraction errors in the included literature. However, this study also has certain limitations: The heterogeneity of the results in the literature included in this study is high, and more high‐quality studies are needed to support the results.

Summarizing the above research results can be drawn: Omentin levels are lower in OSA, which may be a potential marker for OSA diagnosis and treatment. In the future, scholars can increase related research in this area.

## AUTHOR CONTRIBUTIONS

All authors directly participated in the study and have reviewed and approved the final manuscript.

## CONFLICT OF INTEREST STATEMENT

All authors declare that they have no conflict of interest.

## INFORMED CONSENT

Additional informed consent was obtained from all individual participants for whom identifying information is included in this article.

## ETHICS STATEMENT

This article does not contain any studies with human participants or animals performed by any of the authors.

## Data Availability

The data that support the findings of this study are available from the corresponding author upon reasonable request.
